# GPU accelerated voxel-driven forward projection for iterative reconstruction of cone-beam CT

**DOI:** 10.1186/s12938-016-0293-8

**Published:** 2017-01-05

**Authors:** Yi Du, Gongyi Yu, Xincheng Xiang, Xiangang Wang

**Affiliations:** 1 Institute of Nuclear and New Energy Technology, Tsinghua University, Beijing, 100084 China; 2 Department of Engineering, Macquarie University, Sydney, NSW 2109 Australia; 3 Department of Radiation Oncology, Fudan University Shanghai Cancer Center, Shanghai, 200032 China

**Keywords:** Cone-beam CT, GPU, Forward projection

## Abstract

**Background:**

For cone-beam computed tomography (CBCT), which has been playing an important role in clinical applications, iterative reconstruction algorithms are able to provide advantageous image qualities over the classical FDK. However, the computational speed of iterative reconstruction is a notable issue for CBCT, of which the forward projection calculation is one of the most time-consuming components.

**Method and results:**

In this study, the cone-beam forward projection problem using the voxel-driven model is analysed, and a GPU-based acceleration method for CBCT forward projection is proposed with the method rationale and implementation workflow detailed as well. For method validation and evaluation, computational simulations are performed, and the calculation times of different methods are collected. Compared with the benchmark CPU processing time, the proposed method performs effectively in handling the inter-thread interference problem, and an acceleration ratio as high as more than 100 is achieved compared to a single-threaded CPU implementation.

**Conclusion:**

The voxel-driven forward projection calculation for CBCT is highly paralleled by the proposed method, and we believe it will serve as a critical module to develop iterative reconstruction and correction methods for CBCT imaging.

## Background

Cone-beam computed tomography (CBCT) has been advanced to serve as a widely available and commonly used imaging modality in clinical applications, such as dental diagnostics [1], image-guided radiotherapy [2], intraoperative navigation [3], and implant planning [4], and has broadened its usage in new settings, including breast cancer screening and endodontics [5]. However, due to the insufficient data conditioning caused by the circular trajectory, the images of CBCT are susceptible to artefacts, noise and the scatter effect [6]. In order to improve image qualities, increasing research efforts have been directed towards iterative reconstruction algorithms [7].

For iterative methods, most computation time is spent calculating the forward and back projections iteratively, which are indispensable and essential components to model the imaging geometry and X-ray physics. Due to the use of high-resolution flat panel detectors in CBCT, when an iterative reconstruction algorithm is used, the computational load becomes a major issue. Thanks to the advent of graphic processing units (GPUs), massive computation power has been unleashed [8, 9]. In principles, forward and back projections can be generated either in a line-driven or voxel-driven approach. Although both methods deliver the equivalent results with identical theoretical complexities, the compute operations are different in numerical implementation, as shown in Table 1 [9]. When the algorithm shifts from CPUs to GPUs, it is not an intuitive issue because the concurrent threads write data in GPU memories in a scattered manner [10]. The scatter operations potentially cause the *inter*-*thread interference* (or *thread*-*racing*) problem with write hazards. Since gather operations are more efficient than scatter operations for faster memory reads, the strategy of using unmatched projector–backprojector pairs in iterative methods becomes a common solution, as in [11–13] using the ray-driven technique as the projector and the voxel-driven as the backprojector. Nevertheless, Zeng [14] has proved that this bypass scheme will mathematically induce the iterative process to diverge from the true values, and thus matched projector/backprojector pairs are preferred for their mathematical stability and robustness to noise.

**Table 1:** Gather and scatter operations involved in forward and back projection computes

Approach	Forward projection	Back projection
Voxel-driven	Scatter	Gather
Line-driven	Gather	Scatter

Several compute models have been proposed as matched forward/back projector pairs, including distance-driven [15] and separable-footprint approaches [16], and some have been successively GPU-accelerated with specific strategies [17–19]. Among these models, the voxel-driven method is extensively used to perform CBCT forward and back projections for its low complexity. While the voxel-driven backprojection is easy to be GPU-accelerated, due to the nature of scatter operation (as in Table 1), the implementation of its matched forward projector on GPUs is embarrassingly nonparallel, and, to our knowledge, its efficient GPU-based acceleration has never been reported yet.

In this study, a GPU acceleration method is present to calculate voxel-driven forward projections for CBCT iterative reconstruction. This paper is organized as follows: the voxel-driven projection algorithm and the inter-thread interference problem are first investigated in “Voxel-driven model and inter-thread interference study” section; based on the analysis, the proposed GPU acceleration method is detailed in “Combating strategy by optimizing thread-grid allocation” section, with a brief workflow in “Implementation outline” section; as method validation, computational simulations are performed with results given in “Experiment and results” section; some issues are discussed and major conclusions are drawn in “Discussion and conclusion” section.

## Methods

### Voxel-driven model and inter-thread interference study

**Fig. 1:**
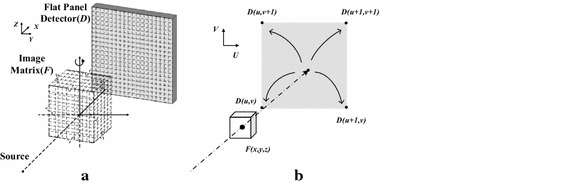
Schematic of CBCT imaging (**a**) and the voxel-driven forward projection algorithm (**b**), where the image voxel value is scattered into the four adjacent detector units

For a typical CBCT scanner, the patient (or scanned object) is kept stationary, and the X-ray source and the flat panel detector are rotating simultaneously around the object in a circular trajectory. To facilitate the mathematical description, the scanned object is discretized as a three-dimensional image matrix, and the flat panel detector as a two-dimensional grid, as in Fig. 1a. In the voxel-driven method, the values of the image matrix are assumed to locate at the centre of each cubic voxel. To generate the two-dimensional forward projections for CBCT through the image matrix, the algorithm can be summarized into three steps: (1) draw a virtual line from the source (*S*) to a voxel centre (*F*(*x*,*y*,*z*)), which represents an X-ray pencil beamlet casting through the voxel; (2) extend the line from the voxel to intersect the detector plane at one point (*U*(*u*,*v*)), which represents the position where the traversal beamlet reaches the flat panel detector; (3) scatter the image value of the voxel into the adjacent detector units as the simplified process of X-ray signal detection, as illustrated in Fig. 1b.

**Fig. 2:**
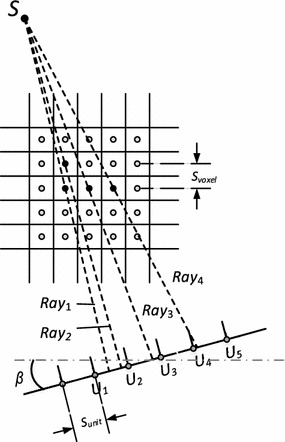
Schematic of the voxel-driven forward projection algorithm for adjacent voxels at β

Conventionally thread grids are allocated to adjacent voxels in axial planes, as reconstructed images are preferred to be displayed in the axial direction. To facilitate the analysis of the inter-thread problem, the three-dimensional forward projection scenario in CBCT is simplified into two-dimension, as illustrated in Fig. 2. The beamlets from the X-ray source (S) go through each voxel and cast onto the detector. For two arbitrary voxels, the distance between their ray-casting intersections on the detector, Δ*u*, can be derived from the imaging geometry relationship as

1 $$\Delta u = \Delta v \cdot F_{g} \cdot \left| {\cos \beta } \right|$$

where *β* is the projection angle, *F*_*g*_ the geometric factor, and Δ*v* the distance between the voxels.

For two neighbouring voxels, Δ*v* is equal to the voxel size, i.e.:

2 $$\Delta v = S_{voxel}$$

In the meantime, we can also rewrite the distance between the ray-casting intersections, Δ*u*, using the detector unit size as:

3 $$\Delta u = S_{unit} \cdot \Delta N$$

where Δ*N* represents the relative distance normalized by the detector unit size *S*_*unit*_.

The geometric factor *F*_*g*_ can be derived according to the imaging geometry and written as

4 $$F_{g} = \frac{SDD}{SVD}$$

where *SVD* stands for the source-to-voxel distance, and *SDD* the source-to-detector distance.

For a typical CBCT, the size of the image voxel is settable according to the user’s choice. Since the highest resolution is usually preferred by radiologists for more image details, the voxel size can be expressed as

5 $$S_{voxel} = \left( {\frac{SDD}{SAD}} \right) \cdot S_{unit}$$

where *SAD* stands for source-to-axis distance.

When we replace the respective terms of Eq. (1) with Eqs. (2)–(5), the distance between the projection intersections of two neighbouring voxels is rewritten as:

6 $$\Delta N = \left( {\frac{SAD}{SVD}} \right) \cdot \left| {\cos \beta } \right|$$

where Δ*N* stands for the relative distance normalized by the detector unit size.

Due to the cone-beam effect in CBCT, the value of the first term, $\left( {\frac{SAD}{SVD}} \right)$, changes along the beamlet, but is always around 1; for the second term, |cos *β*|, it’s always less than or equal to 1.

Meanwhile, in Fig. 2, we can see that for adjacent voxels in the same plane, some of them are cast into adjacent detector grids (as *Ray*_1_ and *Ray*_3_ in Fig. 2), and some into different grids (as in *Ray*_3_ and *Ray*_4_ in Fig. 2). Moreover, for the voxels whose beamlet paths are quite close to each other (as *Ray*_1_ and *Ray*_2_ in Fig. 2), they will be projected into the same detector grids. Note that, since each thread is assigned to each image voxel and each detector grid to each tally address on the GPU, when two voxels are cast into adjacent or identical detector grids, the underlying two threads will try to write data to the same memory address on the GPU simultaneously, which leads to *write hazards*—this is what we call the *inter*-*thread interference* problem, or the *thread racing* problem.

The analysis implies that if the thread grids are allocated to image voxels closely one by one, some threads will race against each other in GPU memory accessing. Unless a specific strategy is taken, this phenomenon is certain to happen and is impossible to avoid. In the meantime, Fig. 2 also shows that if thread grids are allocated in axial planes or *horizontally*, the *worst case* will show up in the central axial plane at all projection angles. However, for the planes above or below the axial plane, the blow of the thread-racing (inter-thread interference) problem is softened because of the cone-beam geometry.

It is noted that although the geometric analysis above is based on the axial plane, because of the symmetry of cone-beam geometry along the central axis, the discussion is also applicative in the vertical planes. Similar conclusions can be drawn when the thread-grid are allocated in the vertical planes.

### Combating strategy by optimizing thread-grid allocation

**Fig. 3:**
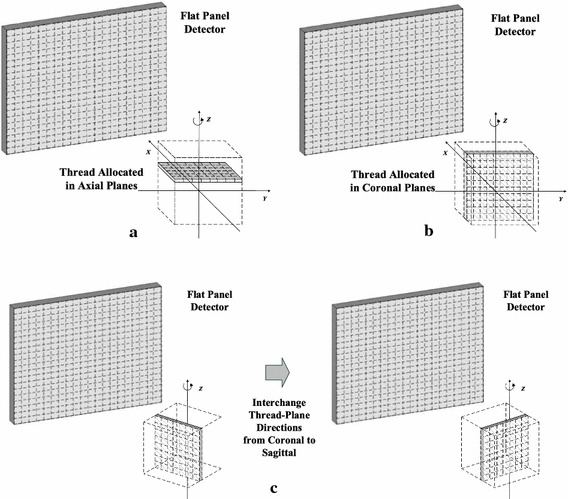
Conventional threads are allocated in *horizontal planes* (**a**). In the proposed method, the threads are allocated in *vertical* (coronal) *planes* (**b**), and the thread-plane direction is interchanged at certain angles from coronal to sagittal (**c**)
- Interchange the thread-plane direction at the critical projection angles
  In fact, as illustrated in Fig. 3c, the worst case of inter-thread interference induced by *Step* (*a*) can be easily solved by interchanging the thread-plane direction from the coronal planes to the sagittal planes at certain projection angles. Here we call the angles for thread-plane direction interchange the *critical angles*. The critical angles are dependent on the imaging and scanner specifications, including *SAD*, *SDD*, and *S*_*unit*_, but can be easily obtained by simulation.
- Serialize the residual interfering threads by atomic operations
  By the two steps above, the thread-racing occurrence frequency can be much decreased. To combat the residual threads that still interfere with each other, we use the GPU-enabling atomic operations to serialize the read-and-write operations among these threads. The mechanism of atomic operations is like an address access lock: at the same moment, only one thread is authorized, and all the others are forced to wait in queue [20].

Based on our discussion, the inter-thread interference phenomenon always come across to a certain degree, which becomes the major hindrance for GPU acceleration. To combat the problem, what we need is a concrete solution to reduce the occurrence frequency to as low as possible and serialize the residual racing threads in the same process. Rising out of the idea that the cone-beam geometry can be utilized to soften the blow of thread racing, we propose a strategy of optimizing the thread-grid allocation to achieve GPU acceleration. The method comprises three key steps:

- Allocate thread grids in the vertical planes (or *vertically*)
  We denote the axial direction as *the horizon direction* (as in Fig. 3a) and the coronal and sagittal directions as *the vertical directions* (as in Fig. 3b). By allocating threads vertically, the thread-racing frequency of the voxels along the same X-ray light path is much decreased. However, as a side-effect, the worst case of inter-thread interference is shifted from the central axial plane at all projection angles to the vertical planes at perpendicular angles to the detector plane, where *β* is equal to 90° or 270°. Then the second step is needed to solve this side-effect problem.

### Implementation outline

The key idea of the acceleration method is described in the above. For reference, the core framework is depicted in the form of pseudo-codes in Table 2. Once the initialization on GPU is completed, the key processes can be implemented as a kernel CUDA function.

**Table 2:**
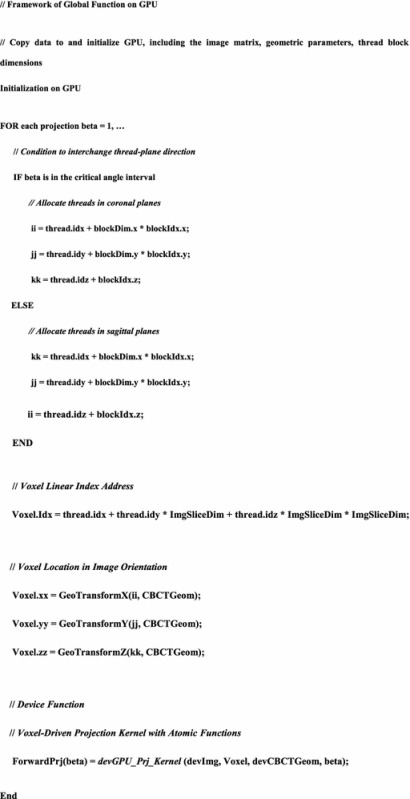
Outline of the GPU acceleration method

## Experiment and results

For method validation, computational simulations are performed using the Shepp-Logan phantom. The simulation scenario specifications are similar to our in-house CBCT scanner geometry [21]: the flat detector panel has 512 × 512 units, and the size of each unit is 0.127 mm; the source-to-axis distance is 80 cm, and the source-to-detector distance 100 cm; projections are calculated over 360° with a 1° interval.

The program is deployed on a Windows Server 2012 workstation with 32-bit single precision. The CPU is Intel Xeon E5-2620, which offers two processors with 12 cores running at a frequency of 2.1 GHz. The GPU is nVidia Tesla K20M. Its capability version number is 3.5, and it has 2696 cores running at a frequency of 0.71 GHz. For comparison, the voxel-driven forward projection generation method is programmed and deployed on the same platform. Since multi-thread parallelization of the voxel-driven forward projection algorithm on CPU also has to deal with the inter-thread interference problem among CPU threads, which is beyond the scope of this study, the algorithm is implemented on a single threaded CPU, and the single threaded running time is recorded as benchmark for performance assessment. Besides, in order to achieve higher accuracy, an 8-subvoxel splitting strategy used: each voxel is first divided 8 cubic subvoxels, and then each subvoxel is forward projected on the detector with 1/8 weight of the father voxel value. Note that the recorded times only account for the process of forward projection kernel excluding the time of transferring data between CPU and GPU.

**Fig. 4:**
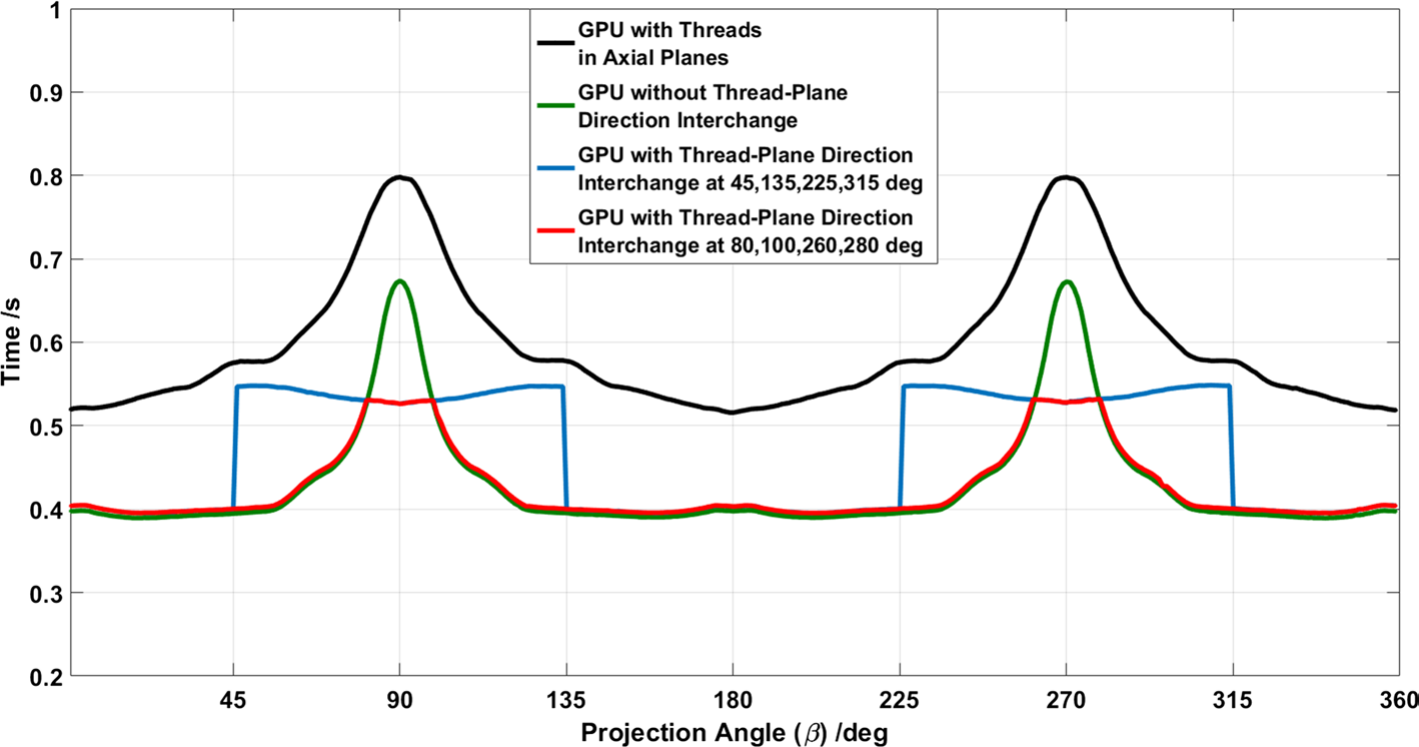
Calculation time curves of different methods: threads are allocated in axial planes and racing threads are solved with atomic operations (*black*); threads are allocated in *vertical planes* and racing threads are solved with atomic operations without thread-plane direction interchange (*green*); threads are allocated in *vertical planes* and racing threads are solved with atomic operations with thread-plane direction interchange at given angles (*blue*); threads are allocated in *vertical planes* and racing threads are solved with atomic operations with thread-plane direction interchange at critical angles (*red*)

To obtain the optimal interchange angles or *critical angles*, we first ran the GPU-enabled programme without the thread plane interchange, and collected the calculation times (green curve in Fig. 4). Then, we interchanged the thread plane at 45°, 135°, 225°, and 315°, and got the new calculation times (blue curve in Fig. 4). When the two temporal curves together were plotted, they intersected with each other, and the intersection angles were the optimal interchange angles. In this scenario, we can see that the optimal interchange angles are 80°, 100°, 260°, and 280°, which are then used as critical angles for thread-plane direction interchange.

As reference, the GPU processing time of using atomic operations to solve all race conditions is plotted as the black curve, and the time without thread-plane interchange is drawn as the green curve in Fig. 4. The computation times of different methods are listed in Table 3, with the CPU computation time as benchmark. We can see that the GPU acceleration ratio of the proposed method is as high as 105.

**Table 3:** Computation efficiency comparison of different methods

Projection frames/image matrix dimension	Method	Total time (s)	Average time (s)	Acceleration ratio
360/512^c^	CPU^a^	360 × 45	45	1
	GPU^b^	214.97	0.597	75.36
	GPU^c^	168.49	0.468	96.15
	GPU^d^	153.499	0.426	105.54

^a^CPU implementation on a single thread, ^b^ GPU acceleration with threads allocated in axial planes, ^c^ GPU acceleration without thread-plane interchange, ^d^ proposed GPU acceleration method

## Discussion and conclusion

As detailed in “Methods” section, the proposed method consists of three key steps. For the first two steps, they are mainly aimed to reduce the inter-thread interference occurrence frequency. From the results in Table 3, we can see that both steps contribute to the calculation acceleration, and Fig. 4 unveils their respective roles: (1) comparing the method of allocating thread grids in axial planes (black curve) and in vertical planes (green curve), we can see the optimization of thread-grid plane can save more than 20% processing time; (2) comparing the method with and without interchanging the thread-grid plane direction, i.e. the red and green curve respectively, we can conclude this operation performs effectively in reducing the peak compute time.

Besides, in Fig. 4, we can see a stair jump effect in the calculation time (as the blue curve) after we interchange the thread grids from coronal planes to sagittal planes. Since the three-dimensional image matrix is stored voxel by voxel in linear memory addresses on GPUs, when a thread is accessing the memory it not only reads the data in the specified address, but also loads the data in adjacent addresses into the GPU cache for possible further usage: this mechanism is what we call *memory coalescing*, which is highly beneficial for fast data accessing [22]. For our method, thread grids are initially bound to voxels that are saved in coalescing addresses. When we interchange the thread-plane direction, the address coalescing condition is corrupted, and data accessing will take more time.

In terms of the critical angles, to investigate their dependence on the CBCT geometric specifications, several scenarios were set up with SAD/SDD ranging from 0.6 to 1. The critical angles were obtained in the same way as in “Experiment and results” section. Only a slight dependence is observed, and the critical angles in different scenarios are fairly close to each other—around 80°, 100°, 260°, and 280°. So we can imply that, from a practical perspective, this set of critical angles performs effectively, and they can be used as empirical values.

In summary, we propose a GPU acceleration method of calculating voxel-driven forward projection for cone-beam CT. The method is composed of three key steps and is easy to implement. The experimental results demonstrate its effectiveness and efficiency in handling the inter-thread interference problem, and a surprising acceleration ratio, as high as 105, has been achieved. It should be noted that the CPU implementation runs on a single thread. A multicore CPU implementation using 6 cores can be accelerated and run faster (for example using OpenMP and streaming SIMD extensions (SSE)), which would reduce the speedup, but certain approach is also required to combat the thread racing problem on CPU.

Besides, using a more sophisticated forward projection method is probably able to achieve improved accuracy. For example, Long et al. [16] proposed a voxel-driven method combining a full voxel model and a detector unit response. In their method, the boundaries of each cubic voxel are first ray-cast onto the detector to generate a polygonal pattern, and then the pattern multiplies a trapezoid/rectangular function to produce the respective forward projection footprint. As discussed in [16], highly realistic projection images can be delivered, but at the expense of tremendously increasing computational complexities compared with the proposed method here. In the meantime, as in [17], it is of scatter operation in nature as well, so special GPU acceleration approaches are also required to combat the thread racing problem (denoted as read-modify-write errors in [17]). Therefore, in some extent, method selection is like a trade-off between approximation and computation complexity, and it all depends on the application requirements.

We believe the proposed acceleration method is probable to serve as a critical module to develop the iterative reconstruction and correction methods for CBCT imaging, as in our case where this method has already been incorporated into our iterative algorithm development platform and working properly [23]. Since the algorithm is programmed for research only, we believe that, with further coding optimization, a higher speedup can be further achieved.

## Abbreviations

GPU: graphic processing unit
CT: computed tomography
CBCT: cone beam computed tomography
SDD: source-to-detector distance
SAD: source-to-axial distance
SVD: source-to-voxel distance
